# Wie viele Patienten mit entzündlich rheumatischen Erkrankungen haben die technischen Voraussetzungen für Videosprechstunden und sind bereit, fachärztliche Visiten so durchzuführen?

**DOI:** 10.1007/s00393-021-01026-y

**Published:** 2021-06-21

**Authors:** X. Baraliakos, F. Alshakaki, B. Bühring, I. Andreica, U. Kiltz, J. Braun

**Affiliations:** Rheumazentrum Ruhrgebiet am Marienhospital Herne, Ruhr-Universität Bochum, Claudiusstr. 45, 44649 Herne, Deutschland

**Keywords:** Videosprechstunde, SARS-CoV-2-Pandemie, Entzündlich rheumatische Erkrankungen, Fachärztliche Visiten, Neue Medien, Video consultation, SARS-CoV‑2 pandemic, Inflammatory rheumatic diseases, Medical specialist visit, New media

## Abstract

**Hintergrund:**

Die aktuell grassierende SARS-CoV-2-Pandemie und begrenzte Kapazitäten in der ambulanten rheumatologischen Versorgung werfen, auch angesichts der digitalen Revolution, Fragen nach möglichen Alternativen zu klinischen Visiten auf. Ob und inwieweit Patienten mit entzündlich rheumatischen Erkrankungen bereit und in der Lage sind, mit den neuen Medien wie etwa Videosprechstunden (VSS) umzugehen, ist unklar.

**Methoden:**

Mitten in der Pandemie wurden im Mai 2020 ambulante Patienten mit einem standardisierten Fragebogen systematisch befragt, um ihre Möglichkeiten und die Bereitschaft für die Teilnahme an VSS zu ermitteln. Der behandelnde Arzt gab an, ob er die Durchführung einer VSS für möglich und auch für sinnvoll hielt.

**Ergebnisse:**

Insgesamt wurden 232 Patienten mit entzündlich rheumatischen Erkrankungen befragt (64,7 % weiblich, mittleres Alter 54,0 ± 15,2 Jahre), seropositive (*n* = 58) und seronegative (*n* = 51) rheumatoide Arthritis (RA), Spondyloarthritis (SpA) (*n* = 77) inklusive der axialen SpA (axSpA) und der Psoriasisarthritis (PsA) sowie Kollagenosen und Vaskulitiden (KoV) (*n* = 46). Die mittlere Krankheitsdauer betrug 5,5 ± 8,2 Jahre, bei 75 Patienten (32,3 %) handelte es sich um eine Erstdiagnose. Die mittlere Krankheitsaktivität (0–10, subjektive Patienteneinschätzung) lag bei 4,7 ± 2,5. Insgesamt wussten 176 Patienten grundsätzlich über die Möglichkeit der Durchführung von VSS Bescheid (75,9 %), und 166 sahen sich technisch in der Lage, daran teilzunehmen (71,6 %), aber nur 131 waren grundsätzlich auch bereit dazu (56,5 %). Die logistische Regressionsanalyse zeigte, dass die Bereitschaft zur Teilnahme an VSS mit zunehmendem Alter abnahm (β = 0,28, *p* = 0,01). Nach ärztlicher Einschätzung wurden VSS bei 161 Patienten aus technischen (69,4 %) und bei 127 aus medizinischen Gründen (54,7 %) prinzipiell für möglich gehalten. Die Durchführung von VSS im Rahmen der Versorgung wurde vom Arzt aber nur bei 76 Patienten (32,8 %) für sinnvoll gehalten.

**Zusammenfassung:**

Nicht alle Patienten können oder wollen an VSS teilnehmen, mit zunehmendem Alter nimmt die Bereitschaft dazu ab. Auch die ärztliche Einschätzung der Sinnhaftigkeit von VSS beschränkte sich auf etwa ein Drittel der befragten Patienten. Dies ist für zukünftige Planungen von VSS zu berücksichtigen.

## Abstract (en)

**Background:**

The currently disseminating severe acute respiratory syndrome coronavirus 2 (SARS-CoV-2) pandemic and limited capacities in outpatient rheumatological care, pose questions about possible alternatives to clinical visits, also in view of the digital revolution. It is unclear whether and to what extent patients with inflammatory rheumatic diseases are willing and in a position to deal with the new media, such as video consultation.

**Methods:**

In the middle of the pandemic in May 2020 outpatients were surveyed using a standardized questionnaire in order to document their possibilities and willingness to participate in a video consultation. The treating physicians were asked whether carrying out a video consultation was considered to be a possible and meaningful option.

**Results:**

Overall, 232 patients with inflammatory rheumatic diseases were surveyed (64.7% female, average age 54.0 ± 15.2 years), seropositive (*n* = 58) and seronegative (*n* = 51) rheumatoid arthritis (RA), spondyloarthritis (SpA, *n* = 77) including axial SpA (axSpA) and psoriatic arthropathy (PsA) as well as collagenosis and vasculitis (CoV, *n* = 46). The mean duration of disease was 5.5 ± 8.2 years, whereby in 75 patients (32.3%) it was the first diagnosis. The mean disease activity (0–10, subjective patient self-estimation) was 4.7 ± 2.5. Overall, 176 patients were basically aware of the possibility to carry out video consultations (75.9%) and 166 considered that they were technically capable to participate (71.6%) but only 131 were principally willing to participate (56.5%). Logistic regression analyses showed that the willingness to participate in video consultations decreased with increasing age (β = 0.28, *p* = 0.01). According to the medical estimation video consultations were thought to be principally possible for 161 patients for technical reasons (69.4%) and for 127 for medical reasons (54.7%); however, a video consultation within the framework of treatment was only considered to be meaningful by the physician for 76 patients (32.8%).

**Conclusion:**

Not all patients can or want to participate in video consultations and the willingness declines with increasing age. The estimation of the meaningfulness of video consultations by physicians was also limited to approximately one third of the patients surveyed. This must be taken into consideration for the future planning of video consultations.

Der Einsatz von Telemedizin hat in den letzten Jahren an Bedeutung gewonnen, und deren Möglichkeiten wurden in Projekten und Studien belegt [1–4]. Einen Überblick über Projekte aus verschiedenen Fachrichtungen gibt das Deutsche Telemedizin-Portal [5]. Die Durchführung von Videosprechstunden (VSS) wird seit mehreren Jahren als Teil moderner Versorgungsstrukturen gefördert [6, 7]. Auch in der Rheumatologie bieten telemedizinische Ansätze neue und spannende Anwendungsmöglichkeiten [2–4, 8]. Da Patienten mit entzündlich rheumatischen Erkrankungen meist chronisch krank sind und zu einem großen Teil ambulant und elektiv betreut werden [9], können telemedizinische Sprechstunden für ausgewählte Patienten eine sinnvolle Ergänzung zur Routineversorgung darstellen.

Durch die Digitale-Gesundheitsanwendungen-Verordnung (DiGAV, [10]) sind neue, zum Teil auch bereits abrechnungsfähige [11, 12] telemedizinische Anwendungsmöglichkeiten, wie z. B. die Nutzung von Apps, entstanden. Ein Überblick über bestehende telemedizinische Versorgungsmöglichkeiten, die aktuelle wissenschaftliche Evidenzlage der Telemedizin in der Rheumatologie einschließlich Chancen sowie Limitationen der Technik wurden vor Kurzem publiziert [2], ebenso eine allgemeine Einschätzung der Deutschen Gesellschaft für Rheumatologie (DGRh, [8]) und der Kommission „Digitale Rheumatologie“ der DGRh [13].

Die Akzeptanz von VSS war bei Ärzten aus unterschiedlichen Gründen allerdings bis vor Kurzem gering [14]. Durch die SARS-CoV-2-Pandemie hat sich im Rahmen des Infektionsschutzgesetzes sowie der verordneten Kontakt- und Mobilitätsbeschränkungen die digitale Kommunikation in der medizinischen Versorgung in vielen Ländern schon deutlich entwickelt [15]. Nach Angaben der Kassenärztlichen Bundesvereinigung (KBV) ist die Zahl der Ärzte, die sich für eine VSS haben registrieren lassen, in kürzester Zeit stark angestiegen [16]. Im Rahmen der vertragsärztlichen Versorgung in Deutschland könnten ca. 20 % aller Hausärzte diese Konsultationsform ihren Patienten bereits anbieten [17]. Neben diesen gehören die Fachinternisten wahrscheinlich zu den Ärzten, für die aufgrund eines hohen Beratungsaufwandes der Einsatz der VSS, gerade auch in der aktuellen Situation der Pandemie, naheliegend ist. Zahlen für die Rheumatologie fehlen aktuell noch, aber auch rheumatologische Einrichtungen in Deutschland könnten von einer digitalen Form der Patientenbetreuung profitieren. Neben den grundsätzlichen bereits gesetzten rechtlichen und abrechnungstechnischen Rahmenbedingungen hat die DGRh kürzlich den möglichen Stellenwert der VSS in der Rheumatologie analysiert und künftige Einsatzmöglichkeiten diskutiert, um die Weiterentwicklung dieser in der Rheumatologie bislang wenig genutzten Versorgungsstruktur zu unterstützen [8].

## Methoden

Das Rheumazentrum Ruhrgebiet ist das größte deutsche Fachkrankenhaus für rheumatische Erkrankungen [18], in dem im Jahr 2019 über 25.000 ambulante Patientenkontakte dokumentiert wurden. Im Mai 2020 wurden, mitten in der SARS-CoV-2-Pandemie, zufällige Patienten aus unserer Ambulanz von einem Facharzt für Rheumatologie befragt, ob sie daran interessiert sind, mit einem zuvor ausgearbeiteten standardisierten Fragebogen systematisch befragt zu werden, um ihre Möglichkeiten und die Bereitschaft für die Teilnahme an VSS zu ermitteln. Neben Patienten- und Krankheitscharakteristika wurden Fragen zur technischen Voraussetzung sowie Bereitschaft zu einer Teilnahme an VSS erhoben. Assessments zur Krankheitsaktivität und körperlichen Funktionsfähigkeit wurden während der klinischen Visite in der Routineversorgung übernommen. Im speziellen wurden folgende Scores erfasst: Disease-activity-Score 28 (DAS-28), Simple disease activity score (SDAI), Funktionsfragebogen Hannover (FFbH), Bath Ankylosing Spondylitis Disease Activity Index (BASDAI), Ankylosing Spondylitis Disease Activity Score (ASDAS), Bath Ankylosing Spondylitis Function Index (BASFI). Die Krankheitsaktivität wurde zusätzlich von allen Patienten als globales Patientenurteil selbst mit einer numerischen Ratingskala (NRS) 0–10 erfasst (0 = keine, 10 = sehr hohe Krankheitsaktivität). Der Arztfragebogen erhob die Ansicht des Arztes, ob dieser die Durchführung einer VSS aus (a) technischen oder (b) medizinischen Gründen für sinnvoll erachtete. Dabei wurden Fragen, die dem Patienten über die technische Ausstattung gestellt worden waren, zur Sicherung der Korrektheit der Angabe erneut gestellt und mit der ursprünglichen Antwort (ja/nein) des Patienten verglichen. Zusätzlich wurde beurteilt (Arzturteil), ob das Krankheitsbild und der allgemeine Gesundheitszustand des Patienten es ermöglichen würden, die gerade stattgehabte Anwesenheitssprechstunde ggf. auch virtuell stattfinden zu lassen.

Eine Patientenselektion wurde nicht vorgenommen.

Die statistische Analyse wurde mittels SPSS v.27 (IBM SPSS Statistics, IBM Deutschland GmbH, Ehningen, Deutschland) durchgeführt. Deskriptive Daten wurden für qualitative Variablen als absolute Zahlen und prozentuale Anteile und für kontinuierliche Variablen als Mittelwerte ± Standardabweichung dargestellt. Vergleiche der Mittelwerte der eingesetzten Scores in unterschiedlichen Subgruppen wurden mittels Mann-Whitney-U-Test durchgeführt. Ein *p*-Wert < 0,05 wurde als statistisch signifikant angesehen. Der Einfluss aller erhobener demografischer und klinischer Parameter auf die Bereitschaft der Patienten zur Teilnahme an der VSS wurde mittels logistischer Regressionsanalyse getestet.

## Ergebnisse

### Patientenkollektiv

Insgesamt wurden 232 Patienten mit entzündlich rheumatischen Erkrankungen befragt (64,7 % weiblich, mittleres Alter 54,0 ± 15,2 Jahre). Darunter waren seropositive (*n* = 58) und seronegative (*n* = 51) rheumatoide Arthritis (RA), Spondyloarthritis (SpA) (*n* = 77) inklusive der axialen SpA (axSpA) und der Psoriasisarthritis (PsA), Kollagenosen und Vaskulitiden (KoV) (*n* = 46). Die mittlere Krankheitsdauer betrug 5,5 ± 8,2 Jahre, wobei 75 Patienten (32,3 %) eine Erstdiagnose erhielten. Die mittlere Krankheitsaktivität (subjektive Patienteneinschätzung) mittels NRS (Skala von 0–10) lag bei 4,7 ± 2,5.

### Ergebnisse der Patientenbefragung

Von allen Patienten waren 176 (75,9 %) allgemein über die Möglichkeit von VSS informiert. Insgesamt 166 Patienten (71,6 %) sahen sich technisch in der Lage, an VSS teilzunehmen, und 131 Patienten (56,5 %) waren auch grundsätzlich bereit dazu. Tab. 1 zeigt die Ergebnisse der Subanalyse für die 176 Patienten, die über die Möglichkeit einer VSS informiert waren, im Vergleich zu denjenigen, die darüber keine Information hatten. In der logistischen Regressionsanalyse wurde nur das zunehmende Alter als Grund für die Abnahme der Bereitschaft zur Teilnahme an VSS ermittelt (β = 0,28, *p* = 0,01). Dabei wurden keine Unterschiede zwischen Patienten mit einer Neudiagnose vs. Patienten mit bekannter Diagnose gesehen.

**Table 1:** 

Gründe zur Teilnahme	Wissen über VSS	
	Ja (*n* = 176)	Nein (*n* = 156)
Technische Möglichkeiten vorhanden	142 (80,7 %)	24 (42,9 %)
Interesse der Patienten	116 (65,9 %)	17 (30,4 %)
Grundsätzliche Bereitschaft zur Teilnahme durch die Patienten	111 (63,1 %)	20 (35,7 %)

### Ergebnisse der Ärztebefragung

Nach ärztlicher Einschätzung wurden VSS bei 161 Patienten aus technischen (69,4 %) und bei 127 aus medizinischen Gründen (54,7 %) prinzipiell für möglich gehalten. Die Durchführung von VSS im Rahmen der Versorgung wurde aber letztlich nur bei 76 Patienten (32,8 %) für sinnvoll gehalten und empfohlen.

Die Analyse zur Bereitschaft (aus Sicht der Patienten) oder die Empfehlung (aus ärztlicher Sicht) für die Teilnahme an der VSS, bezogen auf unterschiedliche krankheitsbezogene Indizes, ist in Tab. 2 dargestellt. Insgesamt wurden ärztlicherseits VSS tendenziell eher bei Patienten mit einem schlechteren Krankheitszustand abgelehnt, wobei dieser Unterschied bei stärkerer Funktionseinschränkung im BASFI und bei Vorliegen von mehr Schmerzen (Globalurteil durch Patienten) signifikant war. Eine ähnliche rein numerische Tendenz wurde für die Bereitschaft zur Teilnahme bei den Patienten beobachtet. Auch hier wurden keine Unterschiede zwischen Patienten mit einer neuen Diagnose vs. Patienten mit einer bekannten Diagnose gesehen.

**Table 2:** 

Erhobener Index (*n* = Patienten)	Patientenangabe			Arztangabe		
	Bereitschaft zur Teilnahme an VSS			VSS empfohlen		
	Ja	Nein	*p*-Wert	Ja	Nein	*p*-Wert
DAS-28 (*n* = 109)	2,8 ± 1,3	2,7 ± 1,2	0,550	2,5 ± 1,1	2,9 ± 1,3	0,251
SDAI (*n* = 109)	13,8 ± 9,6	12,5 ± 7,8	0,709	11,3 ± 8,5	14,5 ± 8,8	0,126
FFbH (%, *n* = 232)	71,3 ± 27,9	69,9 ± 25,3	0,646	74,5 ± 26,6	68,5 ± 26,4	0,198
BASDAI (*n* = 77)	5,1 ± 2,5	4,3 ± 2,1	0,477	4,5 ± 2,6	5,1 ± 2,2	0,343
ASDAS (*n* = 77)	2,8 ± 1,3	2,4 ± 0,9	0,792	2,3 ± 1,3	2,8 ± 1,0	0,108
BASFI (*n* = 77)	4,4 ± 3,0	4,2 ± 2,8	0,895	2,9 ± 2,6	5,2 ± 2,8	*0,045*
PatGU (*n* = 232)	4,5 ± 2,7	4,9 ± 2,2	0,352	3,7 ± 2,7	5,4 ± 2,1	*<* *0,001*

*p-*Wert mittels Mann-Whitney-U-Test. Statistische Signifikanz ist kursiv markiert

## Diskussion

Die Daten unserer Analyse zeigen, dass > 55 % der Patienten in der rheumatologischen Ambulanz einer rheumatologischen Spezialklinik bereit sind, fachärztliche Visiten im Rahmen einer VSS durchzuführen, aber es gibt auch einen ähnlich großen Anteil (45 %), die es nicht sind bzw. die die technischen Voraussetzungen nicht haben. Dass sich hierbei das Alter der Patienten als wichtiger Einflussfaktor herausstellte, ist nicht überraschend. Für jüngere Menschen sind die neuen Medien dagegen bereits so in den Alltag integriert, dass das für die Durchführung von VSS kein Problem zu sein scheint. Zum Verständnis des Gesamtergebnisses kommt hinzu, dass zwar ein Großteil, aber bei Weiten nicht alle Patienten der Meinung sind, dass auch medizinische digitale Anwendungen (Apps) von Vorteil für ihre eigene Gesundheit sein können [19], wobei es wahrscheinlich Unterschiede zwischen der Anwendung einer App und einer direkten Interaktion mit einem Arzt am Bildschirm gibt, die dieses Ergebnis zugunsten der VSS beeinflussen können.

Nichtsdestoweniger war 75 % der Befragten bekannt, dass es VSS gibt, etwa 70 % sahen sich rein technisch dazu in der Lage, und mehr als 50 % der Patienten waren auch bereit, an VSS teilzunehmen.

Der Arzt, der die Befragungen durchführte, hielt allerdings in etwa einem Drittel der Fälle eine Durchführung von VSS bei den Patienten auch für sinnvoll, was in Zeiten von Pandemie und Kontaktreduktion einer letztlich ziemlich klaren Aufforderung gleichkommt, solche Sprechstunden auch anzubieten. Vor allem bei Patienten mit stärkeren Beschwerden (gemessen am Patientenurteil) sowie bei Patienten mit axialer Spondyloarthritis, die höhergradige Funktionseinschränkungen hatten (Daten nicht gezeigt), wurde allerdings die Durchführung von VSS nicht für sinnvoll gehalten. Auch dieses Ergebnis ist nachvollziehbar – davon ausgehend, dass der Arzt bei Patienten, die sich schlecht fühlen oder die erhebliche Funktionseinschränkungen haben, die im Rahmen einer VSS schwerer fassbar wären, eher den direkten Patientenkontakt bevorzugen würde. Insgesamt haben sich sowohl Patienten als auch Ärzte für die VSS entschieden, wenn es den Patienten tendenziell besser ging (bezogen auf die Mittelwerte der Indizes der jeweiligen Erkrankungen).

Zusammenfassend zeigen die hier erhobenen Daten, dass VSS auch in der rheumatologischen Versorgung einen Stellenwert haben können – allerdings unter der Voraussetzung, dass sowohl die technischen Voraussetzungen (bei Patienten und Ärzten) geschaffen werden, als auch, dass die Ärzte in der Lage sind, so akkurat wie möglich die individuelle Patientensituation zu erfassen. Sicherlich wird die VSS eine physisch stattfindende Sprechstunde nicht komplett ersetzen können, v. a. bei Situationen, wo eine Intervention aufgrund lokaler Befunde (z. B. Gelenkinjektion nach entsprechender Untersuchung) notwendig ist. In Zeiten einer Pandemie, aber bei zunehmender Digitalisierung des täglichen Lebens kann die Etablierung einer VSS eine gute Alternative zur physischen Untersuchung sein [15].

Offene Themen dabei sind weiterhin die adäquate Vergütung sowohl der Konsultationsleistung als auch der Bereithaltung der entsprechenden technischen Möglichkeiten sowie organisatorische Hürden, wie z. B. die Rezepterstellung und Übermittlung auf direkt digitalem Wege. Schließlich haben wir noch wenig Erfahrung mit dem Einfluss des empathischen Verhaltens und dem Aufbau einer Arzt-Patienten-Beziehung bei häufiger stattfindenden VSS, verglichen mit den bisher uns bekannten, „konventionellen“ physischen Konsultationen.
